# Actigraphy-based sleep and activity measurements in intensive care unit patients randomized to ramelteon or placebo for delirium prevention

**DOI:** 10.1038/s41598-023-28095-0

**Published:** 2023-01-26

**Authors:** Stuti J. Jaiswal, Samantha R. Spierling Bagsic, Emerson Takata, Biren B. Kamdar, Sonia Ancoli-Israel, Robert L. Owens

**Affiliations:** 1 University of California San Diego School of Medicine, La Jolla, CA USA; 2 The Scripps Research Institute, La Jolla, CA 92037 USA; 3 Scripps Whittier Diabetes Institute, Scripps Health, San Diego, CA USA

**Keywords:** Medical research, Outcomes research

## Abstract

Patients in the ICU often sleep poorly for various reasons, which may predispose to delirium. We previously conducted a clinical trial in which we tested the efficacy of ramelteon, a melatonin-receptor agonist used to treat insomnia, versus placebo, in preventing ICU delirium in patients who underwent elective pulmonary thromboendarterectomy (PTE) surgery. Here we examine sleep, activity, and circadian patterns, measured with actigraphy, to understand changes in these metrics with our intervention and in those with and without delirium. Participants wore wrist actigraphy devices while recovering post-operatively in the ICU. For sleep analysis, we extracted total sleep time and sleep fragmentation metrics over the 22:00 to 06:00 period nightly, and daytime nap duration from the daytime period (0:600 to 22:00) for each participant. For activity analyses, we extracted the following metrics: total daytime activity count (AC), maximum daytime AC, total nighttime AC, and maximum nighttime AC. Next, we performed a nonparametric circadian analysis on ACs over each 24-h day and extracted the following: interdaily stability (IS), intra-daily variability (IV), relative amplitude (RA), and low and high periods of activity (L5 and M10) as well as their start times. These metrics were compared between patients who received ramelteon versus placebo, and between patients who became delirious versus those who did not develop delirium. We additionally made comparisons between groups for daytime and nighttime light levels. No differences in sleep, activity, circadian metrics or light levels were found between drug groups. Delirious patients, when compared to those who were never delirious, had a lower IS (0.35 ± 0.16 vs. 0.47 ± 0.23; *P* = 0.006). Otherewise, no differences in IV, L5, M10, or RA were found between groups. L5 and M10 activity values increased significantly over the post-extubation for the whole cohort. No differences were found for daytime or nighttime light levels between groups. Overall, ramelteon did not impact sleep or circadian metrics in this cohort. Consistent with clinical experience, delirious patients had less inter-daily stability in their rest-activity rhythms. These data suggest that actigraphy might have value for individual assessment of sleep in the ICU, and for determining and detecting the impact of interventions directed at improving sleep and circadian activity rhythms in the ICU.

**Trial registration:** REGISTERED at CLINICALTRIALS.GOV: NCT02691013. Registered on February 24, 2016 by principal investigator, Dr. Robert L. Owens.

## Introduction

Individuals receiving care in the intensive care unit (ICU) suffer from extremely poor sleep^1^. Patients in the ICU demonstrate short sleep duration, a high degree of sleep fragmentation, a decrease in Stage 3 (slow wave) and rapid-eye-movement (REM) sleep, as well as altered circadian rhythmicity^1–4^. Another notable feature of sleep in the ICU is the wide variability in individual sleep and circadian patterns, as well as the different factors that patients identify as disrupting sleep^5^. Poor sleep is clinically relevant as it likely contributes to the development of delirium^6,7^, a common problem^8–10^ with short and long-term sequalae including increased mortality and longer-term neurocognitive decline^11–13^. As such, methods to prevent delirium are needed for hospitalized patients in the ICU. Successful approaches to prevention have often included pharmacologic and non-pharmacologic measures that target sleep in some capacity^14–17^. However, there have been little data on a more personalized approach to sleep assessment and therapies in the hospital setting.

Ramelteon is a synthetic, melatonin-receptor (types 1 and 2) agonist that is FDA-approved for the treatment of insomnia. In the outpatient setting, studies have shown that ramelteon increases total sleep time (magnitude of effect approximately 7 min) and sleep efficiency and decreases subjective sleep latency (magnitude of effect approximately 4 min)^18^. A 2014 study showed that ramelteon prevented delirium in a group of 67 acutely ill hospitalized patients (both on general ward and non-intubated in the ICU), though the mechanism by which this effect was mediated is unknown as no differences in sleep parameters were reported between groups. A subsequent study also demonstrated that ramelteon prevented delirium in an intubated ICU cohort of 88 patients^19^, and in this study, the number of nighttime awakenings was found to be fewer in the ramelteon group compared to placebo. Neither study objectively measured sleep, relying on subjective reporting, medical record review, and observation.

As part of a randomized clinical trial (RCT) testing ramelteon versus placebo effect on delirium in patients recovering in the ICU who underwent elective pulmonary thromboendarterectomy (PTE) surgery, a major cardiothoracic surgery used for the treatment of chronic thromboembolic pulmonary hypertension (CTEPH), we collected objective measures of sleep^20^. Participants wore actigraphy devices post-operatively while recovering in the ICU to address the hypothesis that ramelteon would improve sleep parameters, such as sleep duration and sleep fragmentation, compared to placebo. Actigraphy measures wrist activity and has been shown to correlate well with EEG, the gold standard, measures of sleep^21^. Other studies have also utilized actigraphy as a proxy for basic sleep metrics in the acute care setting^22,23^.

Here we explore the hypothesis that ramelteon would improve actigraphy-based sleep metrics in this cohort, which was a pre-specified outcome in the original study. We further examined basic activity and circadian rest-activity parameters between drug groups to evaluate for differences. Finally, we examined sleep, activity, and circadian rest-activity differences between individuals who developed delirium versus those who did not to address the hypothesis that individuals with delirium would have reduced circadian rest-activity rhythmicity (i.e., objectively measured loss of day-night activity pattern) compared to those who did not develop delirium.

## Methods

### Ethics approval and consent to participate

This study conducted in accordance with the Declaration of Helsinki, was granted approval by the UCSD Human Research Protections Program (#151294). Written, informed consent was obtained from each participant on the night prior to their procedure after a detailed explanation of the risks and benefits of participating was conducted and all participant and family questions were answered. The study was registered at clinicaltrials.gov (NCT 02691013, date of registration: February 24, 2016).

### Parent study

**Figure 1:**
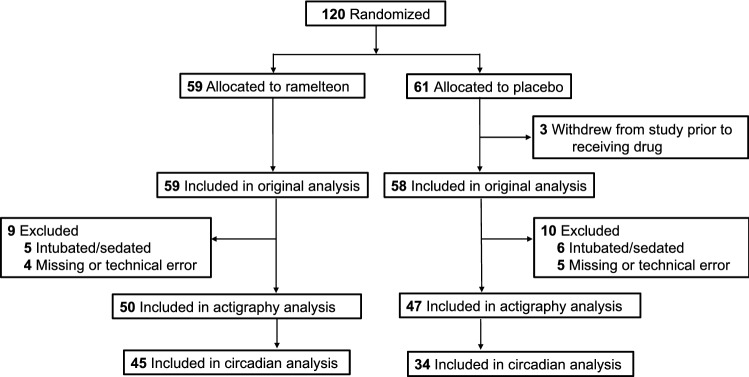
Participant flow diagram. 120 participants were randomized as part of the original clinical trial. There were 97 patients include in the actigraphy analysis presented here.

Detailed methods of the randomized control trial are published elsewhere, and a flow diagram for the current study that also includes patient enrollment from the original study is shown in Fig. 1^20^. Briefly, adults with CTEPH admitted for elective PTE surgery and age ≥ 18 years were approached for enrollment on the night prior to their procedure. Patients undergoing this surgery receive highly protocolized care and are all admitted to the cardiothoracic ICU immediately following their surgeries where they recover under the care of a specialized team of nurses and physicians. Individuals were excluded if they did not speak English, were pregnant, had end-stage liver disease/cirrhosis, or were prescribed fluvoxamine (a selective serotonin reuptake inhibitor known to interact with ramelteon^24^).

After enrollment, participants were randomized in a 4-factor blocked fashion to receive either ramelteon 8 mg or matching placebo each evening at 9 p.m. starting the night prior to surgery (post-operative day-1, or POD-1) and for a maximum of six additional nights (POD 5), or until discharge from the ICU, whichever occurred first. In addition to obtaining daily clinical data, patients were assessed for delirium twice daily using the Confusion Assessment Method ICU (CAM-ICU) through POD 8. Those not scored as CAM-ICU positive (CAM+) on any assessment are termed “never delirious,” while those with at least one CAM + assessment are termed “delirious.” Investigators, subjects, and other clinical care providers remained blinded to drug assignment until trial completion and all data collection was complete.

### Actigraphy

All participants wore an actigraph (Actiwatch Spectrum Plus, Respironics Inc.; Murrysville, PA) beginning after the completion of surgery, placed shortly after their arrival to the ICU. These devices were set to record activity counts in 15 s epochs. Actigraphy devices use accelerometry-based technology to determine activity counts per epoch based on wrist movement. For active patients (e.g., outside the ICU), contiguous periods of inactivity are thought to be rest and/or sleep, and periods of rest can usually be reliably scored by an experienced scorer. However, the activity levels of individuals recovering from surgery in the ICU are very low, and thus manual scoring of the actograms could potentially be unreliable. Thus, we standardized our analysis by defining 22:00 to 06:00 as the nighttime rest period for all individuals, and defined 06:00 to 22:00 as the daytime period. Based on our inpatient experience, the 10 p.m.–6 a.m. period is after nighttime medications are normally administered (9 p.m.), hall lights are dimmed, and is generally the time where rest/sleep is encouraged by bedside nurses. Choosing a “rest interval” was an effort to standardize the rest period and to compare between our randomly allocated groups regarding the amount of sleep captured by the device. We also excluded individuals who were intubated for the entire duration of the study (through POD 8). We did not examine actigraphy data beyond the day of ICU discharge as the participant had reached study completion. If a patient left the ICU before noon, this day was not included. All periods were categorized according to post-extubation day. For example, if a patient was extubated on POD 2, this was considered post-extubation day 0. Actiware software (Respironics Inc.) was utilized to extract data from the devices.

### Actigraphy analysis: nighttime activity and sleep metrics

Actigraphy uses minimal-to-absent activity counts as a proxy for sleep. Generally, a scorer sets a rest period based on sleep diaries when available, or when they think the participant was trying to sleep; here, as explained above, we set the nighttime rest period for all individuals to be between 22:00 and 06:00. The software then uses an algorithm to score each epoch in a rest period as either asleep or awake based on the activity count in that given epoch plus the activity counts in the surrounding epochs. Consecutive epochs scored as sleep make up a sleep bout. The sum of all sleep bouts in a given rest period gives the total sleep duration for that period. Shorter sleep bouts (or an increased number of total bouts in a rest period) are suggestive of more fragmentated or unconsolidated sleep. We collected and aggregated the following activity and sleep metrics for all nighttime rest periods that had no more than 5% of data points missing:

1. *Total nighttime activity count*. Sum total of all activity counts in the nighttime period.
2. *Maximum nighttime activity count*. The maximum activity count recorded in the nighttime period.
3. *Sleep duration.* The total minutes scored as sleep in the given 22:00 to 06:00 period which is equivalent to the sum of minutes within all sleep bouts within this period.
4. *Average sleep bout length.* The average duration in minutes of all the sleep bouts that in the nighttime rest period.
5. *Total number of sleep bouts.* The number of sleep bouts in the nighttime rest period.

### Actigraphy analysis: daytime activity and sleep metrics

We analyzed the daytime period for basic information on activity. We collected and aggregated the following metrics for all daytime periods, categorized according to post-extubation day, that had no more than 5% of data points missing:

1. *Total daytime activity count*. Sum total of all activity counts in the daytime period.
2. *Maximum daytime activity count*. The maximum activity count recorded in the daytime period.
3. *Daytime sleep*. The total minutes scored as sleep in the given 06:00 to 22:00 period which is equivalent to the sum of minutes within all sleep bouts within this period.

### Actigraphy analysis: nonparametric circadian rest-activity analysis

We initially attempted to apply a cosinor analysis to the actigraphy data. However, this analysis showed a very poor cosinor fit (*r*^2^ < 0.01; see Supplemental Methods and Results), and thus we approached the circadian rest-activity rhythms analysis using non-parametric methods to extract the main characteristics of circadian activity rhythms as has been done by others^25^. We note that what is described here refers to a circadian activity pattern based on actigraphy, and not the patients’ biologic circadian rhythm—for example, that which might be measured with longer recording periods or melatonin levels. Raw actigraphy data was categorized by each post-extubation day, using a midnight-to-midnight interval, with days with more than 20% of missing data excluded^26^. Using the nparACT package in the R environment (R v. 4.1.1), we extracted the following metrics for each day (with the exception of interdaily stability, for which there is only one value for each patient) for each patient^27^:

1. *Interdaily stability (IS)* Quantifies the degree of stability of rest-activity rhythms between days, with 0 indicating a lack of rhythm and 1 indicating a perfectly stable rhythm.
2. *Intra-daily variability (IV)* Quantifies the fragmentation of activity-rest periods from 0 to 2, with higher values indicated more fragmentation.
3. *Least 5 (L5) average* Provides the average activity level for the daily sequence of 5 h with the least activity, averaged for all available days.
4. *L5 start hour* Onset of the L5 sequence, indicating time of most restful hours.
5. *Most 10 (M10) average* Provides the average activity level for the for the daily sequence of the most active 10 h, averaged for all available days.
6. *M10 start hour* Onset of the M10 sequence, indicating time of most active hours.
7. *Relative amplitude* Obtained by the following calculation: (M10 − L5)/(L5 + M10). Values ranges from 0 to 1, with a higher value indicating a larger amplitude.

### Actigraphy analysis: light analysis

The actigraph device simultaneously collects white light data (reported in lux) with the activity data. Daytime and nighttime light levels were averaged for each participant based on post-extubation day, and are presented according to group (ramelteon vs. placebo; delirious vs. never delirious).

### Data analysis and statistics

Data for the sleep, activity, and non-parametric circadian rest-activity metrics were extracted for each patient as defined and described above. For all metrics except for IS, we first examined the main effects of both the drug and delirium state independently via linear mixed effects models accounting for the repeated measures among participants and estimated marginal means ± standard errors (SE) were reported. Next, to test for associations over time from extubation, we used linear mixed effects models including either drug or delirium state as well as time (based on post-extubation day) and the interaction of either drug or delirium state with time as fixed effects, and a random effect of subject to account for repeated measures. All mixed effects models were conducted using the lmerTest package^28^ in the R environment. IS was averaged between groups and compared using a linear regression that controlled for the number of days of data. Graphical representations for metrics visualized are shown using boxplots with median and interquartile ranges (IQR) for each group on a given post-extubation day.

## Results

### Participants and baseline characteristics

There were *N* = 117 participants in the original intention to treat analysis (Fig. 1). Eleven individuals were excluded due to being intubated for the duration of the study, and nine were excluded for missing actigraphy data. For these analyses, there were 47 individuals in the placebo group (median 2.0 days of data) and 50 individuals in the ramelteon group (median 2.5 days of data). Baseline characteristics for each drug group is shown in Table 1; overall, no major between-group differences were noted. Of these 97 individuals, 33 developed delirium (at least 1 positive CAM assessment), while 64 did not (no CAM positive assessments).

**Table 1:** Baseline characteristics of patients receiving placebo vs. ramelteon.

Characteristic	Placebo (N = 47)	Ramelteon (N = 50)
Age—mean (SD)	56.6 (15.7)	58.1 (14.7)
Female sex—no. (%)	26 (54.1)	22 (46.0)
Body mass index—mean kg/m^2^ (SD)	32.9 (9.3)	29.6 (8.8)
Charlson comorbidity index—mean (SD)	3.2 (2.0)	3.2 (1.4)
Operating room time—mean min (SD)	521.0 (50.1)	509.4 (84.2)
Circulatory arrest time—mean min (SD)	44.0 (20.0)	42.9 (16.6)
ICU length of stay—median days (IQR)	4.0 (3.0–6.0)	4.0 (3.0–6.5)

### Effect of ramelteon on actigraphically-measured sleep and activity metrics

**Figure 2:**
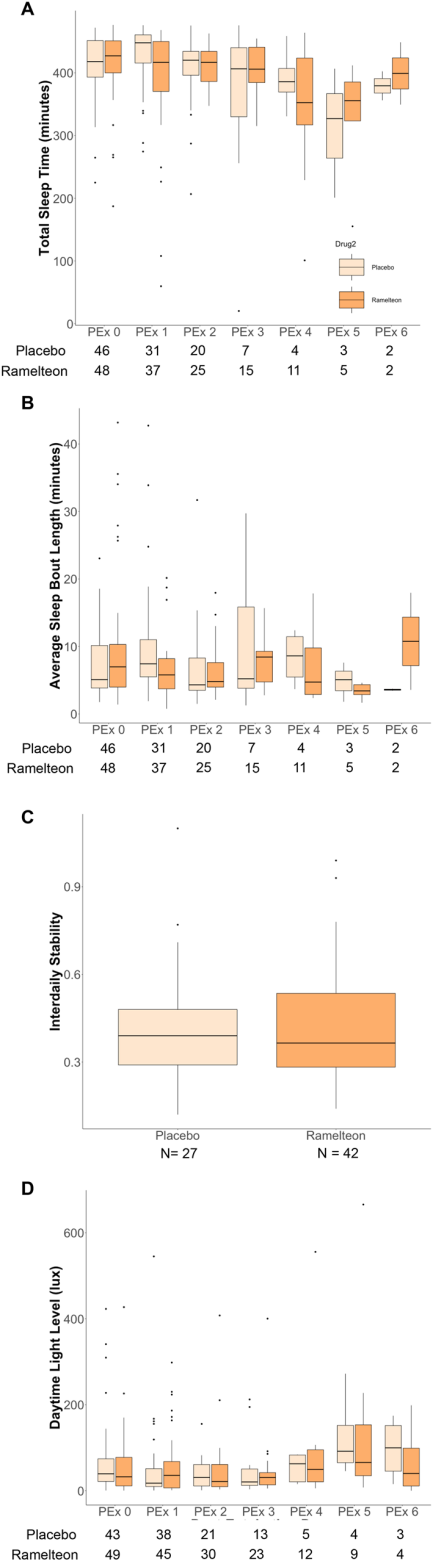
Actigraphy metrics between drug groups. (**A**) Total Sleep Time. Boxplots of total sleep time over the nighttime period between groups based on post-extubation day. (**B**) Sleep Bout Length. Boxplots of average sleep bout length over the nighttime period between groups based on post-extubation day. (**C**) Interdaily Stability. Boxplots of interdaily stability between groups. (**D**) Daytime light exposure. Boxplots of lux levels over the daytime period.

We did not find any differences in sleep or activity metrics aggregated for all periods and for all individuals between active drug and placebo groups (Table 2). Mean estimated nighttime sleep time was 401.0 ± (SE) 8.1 min in the placebo group, and 399.0 ± 7.2 in the ramelteon group (*p* = 0.744). Average sleep bout length was relatively short, 8.6 ± 0.9 min in the placebo group and 7.8 ± 0.8 min in the placebo group (*p* = 0.991). When we examined sleep and activity metrics based on post-extubation day, we also did not find between-drug group differences (Table 2). Total sleep time at night (shown in Fig. 2A along with average sleep bout length in Fig. 2B) was found to decrease significantly over the post-extubation period with an estimated decrease of 12.6 ± (SE) 4.7 min (*p* = 0.008) per day. Daytime sleep also decreased over the post-extubation period (Table 2). Total daytime activity count, maximum daytime activity count, and total nighttime activity count increased significantly over the post extubation period (Table 2).

**Table 2:** Actigraphy data in patients receiving placebo vs. ramelteon.

Characteristic	Placebo (N = 47)	Ramelteon (N = 50)		*P* value	Change over time	*P* value	Drug × time	*P* value
Days analyzed—median (IQR)	2 (1–2)	2 (1–4)						
Nighttime activity and sleep metrics								
Total nighttime activity count—mean count (SE)	5,987 (684)	5535 (618)		0.604	985.3 (386.2)	0.011*	106.6 (486.5)	0.827
Maximum nighttime activity count—mean count (SE)	155 (12.4)	122 (11.2)		0.169	12.3 (7.0)	0.080	0.5 (8.9)	0.958
Total sleep time—mean minutes (SE)	401.0 (8.1)	399.0 (7.2)		0.744	− 12.6 (4.7)	< 0.001***	1.2 (6.0)	0.842
Sleep bouts—mean number (SE)	75.6 (5.2)	76.4 (4.7)		0.976	− 1.2 (3.0)	0.687	0.2 (3.8)	0.959
Average sleep bout length—mean minutes (SE)	8.6 (0.9)	7.8 (0.8)		0.991	− 0.2 (0.6)	0.689	− 0.4 (0.7)	0.592
Nighttime light level (lux)^a^—median (IQR)	1.9 (5.9)	2.2 (5.7)		0.880	N/A	N/A	N/A	N/A
Daytime activity and sleep metrics								
Total daytime activity count—mean count (SE)	20,637 (2,014)	19,189 (1,830)		0.636	4860 (842)	< 0.001***	− 1199 (1053)	0.256
Maximum daytime activity count—mean count (SE)	200.0 (11.1)	190.0 (10.0)		0.741	18.5 (5.4)	< 0.001***	− 7.5 (6.7)	0.266
Daytime sleep—mean minutes (SE)	637.0 (23.7)	644 (21.6)		0.663	− 76.4 (9.4)	< 0.001***	− 4.3 (11.8)	0.718
Daytime light level (lux)^a^—median (IQR)	40.2 (61.9)	48.1 (68.2)		0.346	N/A	N/A	N/A	N/A

Circadian rest-activity rhythm analysis	Placebo (N = 34)	Ramelteon (N = 45)						
IS^b^—mean (SD)	0.42 (0.21)	0.43 (0.21)		0.427	N/A	N/A	N/A	N/A
IV—mean (SE)	1.38 (0.14)	1.41 (0.10)		0.504	1.9 × 10^–3^ (4.3 × 10^–2^)	0.966	1.1 × 10^–2^ (5.5 × 10^–2^)	0.837
L5—mean activity (SE)	3.06 (0.37)	1.79 (0.26)		0.786	0.63 (0.11)	< 0.001***	− 0.38 (0.14)	0.008**
M10—mean activity (SE)	15.4 (1.53)	10.7 (1.10)		0.853	2.97 (0.45)	< 0.001***	− 1.42 (0.57)	0.013*
RA—mean (SD)	0.69 (0.05)	0.71 (0.04)		0.756	− 6.3 × 10^–3^ (1.7 × 10^–2^)	0.703	9.7 × 10^–3^ (2.1 × 10^–2^)	0.642

^a^Nighttime and daytime light levels between groups were compared using Mann–Whitney test.

^b^Interdaily stability was compared using linear regression while all other metrics were compared using linear mixed effects model.

### Effect of ramelteon on circadian rest-activity rhythms

This analysis consisted of 79 individuals and 180 days of data. Table 2 describes results for the nonparametric circadian rest-activity rhythm analysis. Ramelteon did not appear to influence rest-activity rhythm metrics between groups (Table 2). Overall, the L5 and M10 (both of which represent levels of activity) increased significantly over the post-extubation period. We did also note that there was an interaction between drug and post-extubation day that showed a small but signficant decrease in activity with ramelteon over time in the ICU for both L5 and M10 (Table 2). IS (Fig. 2C), IV and RA appeared unaffected by post-extubation day, and no drug by post-extubation day interaction was found for these metrics. The estimated marginal mean for the L5 start time was 08:55 (± 172 min) in the placebo group and 13:32 (± 125 min) in the ramelteon group, but were not significantly different (*p* = 0.971). Similarly, M10 start time was 10:48 (± 78 min) for placebo and 11:18 (± 55 min) for ramelton, but was not different between gorups (*p* = 0.856) Fig. 2D shows daytime light levels between drug groups, but both daytime and nighttime light levels were not found to be different between placebo or ramelteon.

### Actigraphically-measured sleep and activity metrics in delirious vs. never delirious individuals

**Figure 3:**
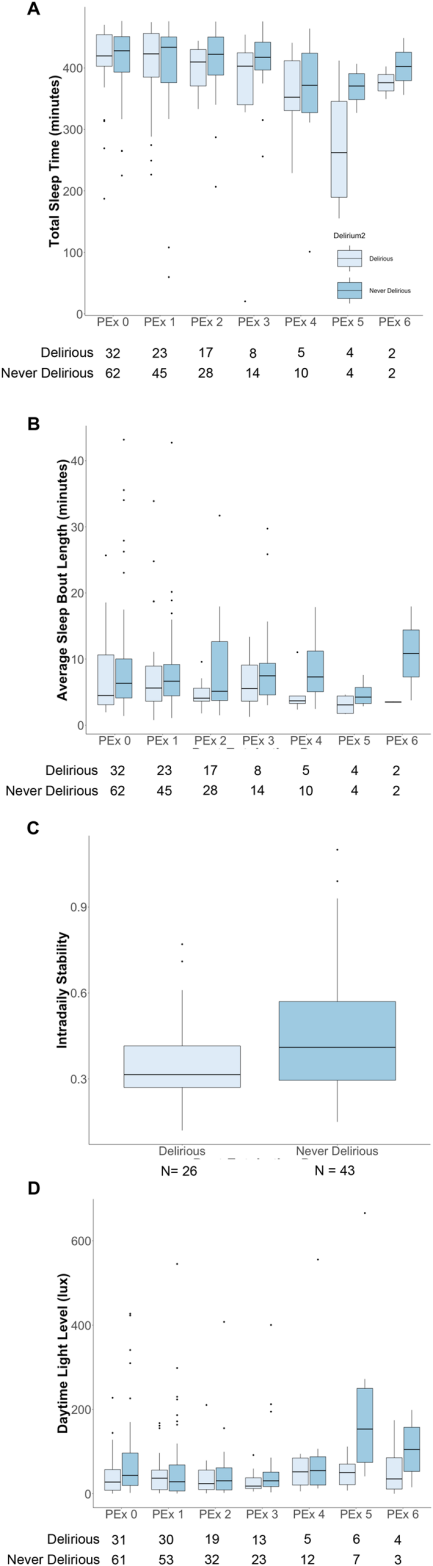
Actigraphy metrics between drug groups. (**A**) Total sleep time. Boxplots of total sleep time over the nighttime period between groups based on post-extubation day. (**B**) Sleep Bout Length. Boxplots of average sleep bout length over the nighttime period between groups based on post-extubation day. (**C**) Interdaily Stability. Boxplots of overall interdaily stability between groups. (**D**) Daytime light exposure. Boxplots of lux levels over the daytime period.

Aggregated data showed that average sleep time during the nighttime rest period (Fig. 3A) was 394.0 ± 8.9 min in delirious patients and 404.0 ± 6.6 min in those who never developed delirium (*p* = 0.619). Average sleep bout length (Fig. 3B) was 8.9 ± 0.7 min in those who were never delirious and 6.8 ± 0.9 min in patients who developed delirium (*p* = 0.584). Other sleep and activity metrics were similar between groups (Table 3) except for maximum daytime activity count, which was found to be significantly lower in delirious individuals (170 ± 12.0 activity counts per epoch vs. 206.0 ± 9.1 activity counts per epoch, *p* = 0.028). Similar to when comparing drug groups, we found decreased sleep time over the post-extubation period and increasing total daytime activity count. Total nighttime activity count and maximum nightime activity count similarly increased over the post-extubation period (Table 3).

**Table 3:** Actigraphy data in never delirious vs. delirious patients.

Characteristic	Never delirious (N = 64)	Delirious (N = 33)	P-value	Change over time	P-value	Delirium × time	P-value
Days analyzed—median (IQR)	2 (1–3)	2 (1–3.25)					
Nighttime activity and sleep metrics							
Total nighttime activity count—mean count (SE)	5,212 (555)	6,624	0.613	657.7 (305.1)	0.032*	888.1 (471.0)	0.061
Maximum nighttime activity count—mean count (SE)	143 (10.4)	127 (14.1)	0.342	10.7 (5.6)	0.058*	3.2 (8.7)	0.704
Total sleep time**—**mean minutes (SE)	404.0 (6.6)	394.0 (8.9)	0.619	− 8.6 (3.7)	0.023*	− 7.6 (5.8)	0.190
Sleep bouts—mean number (SE)	71.5 (4.2)	84.5 (5.7)	0.303	− 1.5 (2.4)	0.533	0.8 (3.7)	0.836
Average sleep bout length—mean minutes (SE)	8.9 (0.7)	6.8 (0.9)	0.584	− 0.3 (0.5)	0.549	− 0.4 (0.7)	0.535
Nighttime light level (lux)^a^—median (IQR)	2.0 (5.0)	1.9 (7.3)	0.864	N/A	N/A	N/A	N/A
Daytime activity and sleep metrics							
Total activity count/24 h—mean count (SE)	21,440 (1674)	16,889 (2220)	0.534	4459.3 (647.4)	< 0.001***	− 831.2 (1038.2)	0.424
Maximum activity count—mean count (SE)	206.0 (9.1)	170.0 (12.0)	0.028*	12.2 (4.1)	< 0.001***	5.0 (6.6)	0.452
Daytime sleep—mean minutes (SE)	625.0 (19.9)	670 (26.5)	0.151	− 77.1 (7.2)	< 0.001***	− 6.8 (11.7)	0.560
Daytime light level (lux)^a^—median (IQR)	50.8 (87.1)	36.6 (50.7)	0.066	N/A	N/A	N/A	N/A

Circadian rest-activity rhythm analysis	Never Delirious (N = 51)	Delirious (N = 28)					
IS^b^—mean (SD)	0.47 (0.23)	0.35 (0.16)	0.006**	N/A	N/A	N/A	N/A
IV—mean (SE)	1.33 (0.10)	1.51 (0.13)	0.116	− 4.1 × 10^–2^ (3.3 × 10^–2^)	0.215	9.7 × 10^–2^ (5.3 × 10^–2^)	0.069
L5—mean activity (SE)	1.92 (0.27)	2.70 (0.35)	0.621	0.28 (0.09)	0.002**	0.26 (0.14)	0.067
M10—mean activity (SE)	13.1 (1.15)	10.8 (1.46)	0.809	2.26 (0.35)	< 0.001***	− 0.59 (0.57)	0.297
RA—mean (SE)	0.74 (0.04)	0.65 (0.05)	0.389	− 1.1 × 10^–2^ (1.3 × 10^–2^)	0.389	2.8 × 10^–3=2^ (2.0 × 10^–2^)	0.169

^a^Nighttime and daytime light levels between groups were compared using Mann–Whitney test.

^b^Interdaily stability was compared using linear regression while all other metrics were compared using linear mixed effects model.

### Circadian rest-activity rhythms in delirious vs. never delirious patients

We found that delirious patients had a lower IS (Fig. 3C) compared to those who were never delirious (0.35 ± 0.16 vs. 0.47 ± 0.23; *p* = 0.006. No differences in L5, M10, or RA were found between groups (Table 3). Similar to findings between drug groups, the L5 and M10 activity values increased significantly over the post-extubation period (Table 3). The estimated marginal mean for the L5 start time was 10:48 (± 88 min) for delirious patients compared to 13:32 (± 69 min) for those never delirious (*p* = 0.499). M10 start time was 11:54 (± 73 min) compared to 10:35 (± 57 min) for those never delirious (*p* = 0.643) Daytime lux levels for each post-extubation day are shown in Fig. 3D. No differences were found for daytime or nighttime light levels between groups (Table 3).

## Discussion

We present several important findings in this study. First, ramelteon, as compared to placebo, did not have an impact on total sleep time, sleep fragmentation, or circadian parameters as measured by actigraphy in this ICU cohort. Those who received ramelteon had a lower activity level over the post-extubation ICU period compared to placebo, though other total activity measures seemed similar. Second, overall circadian fit was low for the whole cohort. Finally, we found that delirious patients had a lower stability in their rest-activity patterns compared to those who did not become delirious. Importantly, our study associated actigraphy data with clinical outcomes, and suggests that actigraphy and similar technologies could be useful in determining sleep, activity, and circadian differences between groups of individuals, even in the ICU.

### Effect of ramelteon on sleep

While some (though not all) studies have shown ramelteon to reduce the incidence of delirium^17,19,29,30^, we note that our original study did not show this benefit in post-operative PTE patients^20^. Broadly, the mechanism of action of ramelteon, which acts on the melatonin receptors, is thought to be improved sleep or maintenance of the circadian rhythm. In the ICU, circadian rhythm is often phase delayed (with acrophase occurring later in the day), or a reduced amplitude is noted. Furthermore, delirium has previously been associated with and attributed to impaired circadian rhythmicity^31^. Based on our objective measurements reported here, ramelteon may not have improved rates of delirium as it did not improve sleep or circadian activity rhythms in this specific cohort. In contrast, Nishikimi et al. found decreased subjective/observed nighttime awakenings (i.e., less fragmented sleep) in the ramelteon group compared to placebo in the 2018 study where ramelteon was associated with less delirium^19^.Another possibility is that actigraphy is not sensitive enough in an ICU population to record improvements in sleep duration or fragmentation. However, observed nighttime awakenings can also be inaccurate and actigraphy is sensitive in other populations with low activity levels, such as nursing home patients^32–35^. Notably, the L5, M10, and other activity metrics appeared lower over the post-extubation period in those who received ramelteon, raising the possibility of a daytime sedating side effect. However, in our original study, we did not find any differences in RASS scores between drug groups^20^.

Given the number of studies suggesting that efforts aimed at improving sleep reduce delirium^7,16,36^, objective measurements of sleep such as these are needed to help confirm mechanism and to identify the most efficient/parsimonious set of interventions (both pharmacological and non-pharmacological) to improve sleep in the ICU.

### Sleep and light in the ICU

We note that, consistent with other studies, sleep in the ICU was quite poor at night. Assuming actigraphy may overestimate sleep time in the ICU, especially for those moving little after major surgery, we note that sleep time decreased each day post-extubation. This decrease is probably due to increased activity/movement that helps better estimate sleep duration (i.e. less time thought to be sleep due to inactivity). Additionally, sleep fragmentation as assessed by sleep bout length remained high throughout. By the end of the ICU stay, total nighttime sleep duration was only about 350 min per patient. Regarding the light data, while we found no between-group differences, we did find that the light levels were very low (generally below 100 lx). Low light levels are desirable at nighttime to help with sleep (high light levels at nighttime can suppress melatonin production, which is important for the maintenance of sleep), but should be much higher during the daytime as it is a major circadian rhythm cue (zeitgeber). This suggests a potential area of improvement for in critical care medicine.

### Sleep and delirium

We and others have previously shown that sleep fragmentation is associated with delirium and cognitive decline, whereas total sleep duration is often similar in those with and without delirium^37–39^. Sleep bout length was shorter in those with delirium here as well, suggesting more sleep fragmentation in this group, but the difference did not reach statistical significance. Given that one of the signs/symptoms of delirium is loss of the day/night cycle (for example used in the ICU Delirium Screening Checklist [ICDSC]), it is not surprising perhaps that we found a less robust rest-activity rhythm in those with delirium. However, it is promising that even though the overall level of activity of this cohort was low, we were still able to see differences between the groups. This suggests that actigraphy, and other tools that use similar technology, may become useful in assessing and treating delirium in the hospital by evaluating individuals’ sleep, activity, and circadian patterns in the acute care setting. Others have also used day vs. night activity patterns to discriminate delirium from no delirium^40^.

### Actigraphy in the ICU

Actigraphy, which relies on movements for its measurements, is not a replacement for polysomnography, particularly in the ICU, where medication, acute illness, and critical illness myopathy can all contribute to decreased activity. However, actigraphy does have benefits over polysomnography in that it is less cumbersome, data can be collected passively for long periods of time and over 24-h periods, and data collection is not limited to information about sleep. Furthermore, more recent digital devices use technology based on actigraphy, and many of these digital devices may be key in improving our approach to personalized medicine in the acute care setting^41^. Our study helps demonstrate that sleep and activity differences between groups of individuals can be assessed^42,43^. Specifically, our findings here show that there are detectable actigraphic changes in delirious patients, including a diminished circadian activity rhythmicity and changes in activity patterns, both of which are consistent with prior studies. We have previously shown in general ward patients that an individuals’ actigraphic signature may change prior to the onset of delirium^44^. Thus, actigraphy, and possibly other newer digital devices, could have a role the assessment and treatment of sleep deprivation and delirium in the critical care setting^45^. In particular, actigraphy may help individualize therapy for improved sleep in several ways. If the major abnormality appears to be sleep fragmentation, efforts to investigate causes can be undertaken. Such underlying causes might be patient specific (e.g. restless leg syndrome)^46^, due to ventilator-patient dyssynchrony, or due to the ICU environment—each of these would be managed differently. Similarly, melatonin and light therapy are typically used to manipulate the circadian rhythm. However, interventions need to be timed appropriately or they may worsen the problem. For example, early morning light delivered before the core body temperature nadir in a person with circadian phase delay might cause further circadian delay. Understanding the patient’s inherent rhythm, as determined by actigraphy, would enable personalized behavioral or pharmacological treatments.

### Limitations

As above, we did not use PSG. We restricted our analysis to days off mechanical ventilation. However, in our cohort, the majority of patients were on a ventilator for less than 24 h, and for most subjects (72%) we therefore analyzed at least 2 days’ worth of data. We did not confirm that the circadian activity rhythm measured by actigraphy reflected other markers of the endogenous circadian rhythm such as core body temperature or serum or urine melatonin measurements. Nevertheless, we emphasize that the circadian differences we saw had biological plausibility (reflected expected changes with administration of a melatonin agonist) and/or reflected clinical differences between groups. Furthermore, this was an exploratory analysis of rest-activity rhythms in the ICU, and resulting analyses were underpowered to detect significant effects. Given the high variability of multiple variables (including activity, light patterns, days studied), these data should serve to inform larger, powered studies in this setting and generate future research hypotheses. Finally, the lack of significant findings in our analysis could have been limited by a shorter recording time (~ 2–3 days of recordings per individual) which may not be adequate to fully assess circadian rhythms as at least 72 h of recording are usually suggested.

## Conclusion

Using objective measurements, we found that the use of ramelteon did not improve sleep duration or circadian rest-activity rhythms. Differences in interdaily stability were seen in those with and without delirium. Further studies are warranted to determine the best uses of actigraphy in the ICU setting.

## Supplementary Information

Supplementary Information.

## Data Availability

Datasets generated from this study are available upon reasonable request to Dr. Owens (rowens@health.ucsd.edu).
