# Supplementary material for: Actigraphy-based sleep and activity measurements in intensive care unit patients randomized to ramelteon or placebo for delirium prevention
Source: Sci Rep. 2023 Jan 26;13:1450. doi: 10.1038/s41598-023-28095-0 (PMC9879948; doi:10.1038/s41598-023-28095-0)
Supplement: Supplementary file 1 — Supplementary Information. [file 41598_2023_28095_MOESM1_ESM.docx]

**SUPPLEMENTAL METHODS AND RESULTS**

**SUPPLEMENTAL METHODS**

**Actigraphy analysis - cosinor analysis.** Raw actigraphy data was categorized by each post-extubation day, using a midnight-to-midnight interval, with days with more than 20% of missing data excluded.^25^ Using the cosinor2 package^26^ in the R environment (R v. 4.1.1),^27^ a cosinor regression model was applied to each day of data using a period of 24 hours with one-minute epochs. We extracted the following metrics for each day for each patient (**Figure 2A**)^21^:

1. *MESOR.* Mean activity count per minute*.*
2. *Amplitude.* Vertical distance (in activity count) from the MESOR to the peak of the cosine wave.
3. *Acrophase*. Time of the peak of activity, using a 24-hour clock, on the cosine wave.
4. *R^2^*. Goodness of fit value from the regression model.

**Data analysis and statistics.** For all metrics, we examined the main effects of both the drug and delirium state independently via linear mixed effects models accounting for the repeated measures among participants and estimated marginal means +/- standard errors (SE) were reported. All mixed effects models were conducted using the lmerTest package^28^ in the R environment.

***Supplemental Figure 1.*** ***Cosinor analysis and examples. A. Cosinor metrics.*** *Example of a cosinor curve with pertinent circadian metrics.* ***B-D. Cosinor regression examples.*** *Panels show examples of one day of raw actigraphy data with the associated cosinor regression line. Examples are shown for participants receiving placebo (Panels B and D), ramelteon (Panel C), and for a participant who did not develop delirium (Panel C) and for one who did (Panel D).*

**SUPPLEMENTAL RESULTS**

Please see Supplemental Table 1 for a summary of the results from the cosinor analysis that was applied to the actigraphy data. Overall, we note a poor cosinor fit in both groups based on R^2^ values. Figures 2B-D contain raw data examples of actigraphy recordings for patients within our cohort.

| Supplemental Table 1. Circadian rest-activity rhythm analysis | |  |  |
| --- | --- | --- | --- |
| *Drug comparison* |  |  |  |
|  | *Placebo*  *(N=34)* | *Ramelteon*  *(N=45)* |  |
| Acrophase – hour (SE) | 14.4 (0.5) | 14.0 (0.4) | 0.087 |
| Amplitude – activity count (SE) | 13.7 (1.8) | 10.9 (1.5) | 0.542 |
| MESOR – activity count (SE) | 25.6 (2.6) | 19.7 (2.2) | 0.789 |
| Fit (r^2^) (SE) | 4.6 x 10^-2^ (5.5 x 10^-3^) | 3.7 x 10^-2^ (4.3 x 10^-3^) | 0.215 |
| Light Acrophase – hour (SE) | 13.9 (0.4) | 14.1 (0.3) | 0.351 |
|  |  |  |  |
|  |  |  |  |
| *Delirium comparison* |  |  |  |
|  | *Never Delirious*  *(N=51)* | *Delirious*  *(N=28)* |  |
| Acrophase – hour (SE) | 14.3 (0.5) | 14.2 (0.6) | 0.756 |
| Amplitude – activity count (SE) | 12.6 (1.4) | 10.9 (1.9) | 0.490 |
| MESOR – activity count (SE) | 22.0 (2.1) | 21.2 (2.8) | 0.954 |
| Fit (r^2^) – (SE) | 4.1 x 10^-2^ (4.2 x 10^-3^) | 4.1 x 10^-2^ (5.6 x 10^-3^) | 0.084 |
| Light acrophase – hour (SE) | 14.2 (0.3) | 13.8 (0.4) | 0.525 |
